# Evaluation of Adhesive Joints Using Ultrasonic Rayleigh Waves

**DOI:** 10.3390/ma17061367

**Published:** 2024-03-16

**Authors:** Jakub Kowalczyk, Dariusz Ulbrich

**Affiliations:** Faculty of Civil and Transport Engineering, Institute of Machines and Motor Vehicles, Poznan University of Technology, 60-965 Poznan, Poland; dariusz.ulbrich@put.poznan.pl

**Keywords:** adhesive joints, non-destructive testing, ultrasound, Rayleigh wave

## Abstract

Adhesive joints are non-separable connections that are used in numerous ways in vehicle construction, particularly in buses. The widespread use of adhesive joints makes it necessary to assess their quality, especially under production conditions. The main goal of this study was to develop a mathematical model to estimate the width of the adhesive path in a plywood-adhesive-closed-profile joint based on selected parameters of the ultrasonic surface wave. A digital ultrasonic flaw detector and Rayleigh wave probes were applied. The test involved evaluating different widths of hybrid adhesive and two-component epoxy adhesive. The tests were conducted on a steel profile from a bus construction. The attenuation of the ultrasonic waves on the steel profile (0.026 db/mm) and the adhesive (0.264 dB/mm) was determined. A one-size-fits-all model for estimating adhesive path width for specific conditions is proposed.

## 1. Introduction

Testing the reliability of technical objects, including joints, is the subject of many articles [1,2,3] and constitutes the basis for improving manufactured machines and vehicles. One of the objects of reliability research is adhesive joints, widely used in every field of industry. Adhesives have numerous applications in aerospace, electronics, automotive and building construction, as well as branches of medicine [4,5,6]. In addition, adhesives are used in light industry, such as toys, packaging and books [7]. Due to their properties, adhesives are widely used for production, as well as repairing machine and equipment components. Adhesive joints have numerous advantages. The main advantages are the sealing of the structure and the favorable, uniform distribution of stresses, as well as the reduction of production costs. The main limitations in the use of adhesive joints, in particular, are the limited shelf life of adhesives, the need for proper surface preparation, the difficulty in assessing the quality of the shaped joint and the relatively low resistance to aging [8]. The strength of an adhesive joint is determined by both the phenomena of adhesion and cohesion.

**Figure 1:**
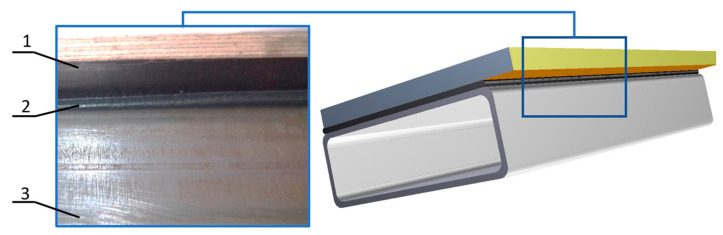
Bus frame with bonded floor plywood: 1—plywood, 2—adhesive, 3—steel frame.

A particularly significant share of adhesive joints is observed in the automotive industry [9,10,11]. These joints are used in numerous applications in the manufacturing stage of cars, vans, trucks and buses (urban two axle, articulated and tourist buses). In the construction of passenger vehicles, bonding is used for fixing brake pad linings, joining windows and elements of the door, engine covers and the body panels. An equally significant share of adhesives in vehicle construction is observed in the production of buses [12]. During their construction, in addition to the parts mentioned above in passenger vehicles, the floor is also bonded to the frame, along with floor carpeting, plating, and side panels to the vehicle frame. Additionally, the front directional panel cover and rear wall, fuel filler caps and roof plating are bonded [13,14]. Such extensive use of bonding in the construction of buses is due to their considerable size and the vibrations generated during operation, which are well damped by adhesives. Adhesive joints in vehicle construction are often combined with welded [15], spot welded [16] or shaped joints, such as clinching [9]. Combining different joining methods speeds up production and increases the mechanical strength of the joint. A cross-section of the floor used in a bus construction manufactured using adhesive bonding is shown in Figure 1.

Adhesive joints are well known, but widespread use requires the evaluation of their quality. Quality assessment using destructive and non-destructive methods can be carried out. The first group of methods of testing adhesive joints are methods that allow us to determine the quality of the joint, but at the same time cause its destruction. These methods are relatively well understood and described in the literature [17,18,19]. The main methods of destructive evaluation of bonded joints are mechanical strength tests. The most widely used destructive method of testing adhesive joints is the determination of their shear and tensile strengths [20]. This group of tests allows the evaluation of both the properties of the adhesives and the appropriateness of the adhesive technology (surface preparation, adhesive preparation, conditions for manufacturing the joint and others).

In contrast to destructive methods for evaluating adhesive joints, non-destructive techniques are utilized. Non-destructive methods used for the estimation of adhesive joints include—first of all—such methods as visual [21], ultrasonic [22,23], acoustic emission [24] and thermographic [25], as well as terahertz [26] and synchrotron X-ray technology which are also widely used in non-destructive testing [27]. Among these methods, ultrasound, which is based on the phenomenon of the reflection and refraction of waves with frequencies above 16 kHz, is often used. With regard to the examination technique, one can distinguish between the echo method and the transmission method. The echo method requires the use of a single ultrasound transducer, while the transmission method uses two ultrasound transducers—a transmitting and a receiving one. Ultrasonic testing uses different types of waves, such as longitudinal waves, transverse waves and plate waves [28,29]. Ultrasound is used to detect defects in adhesive joints [30], such as kissing bonds [31,32]. Samaitis et al. [33] conducted tests on aluminum adhesive joints of varying quality (high quality joints and defective joints with contaminated surfaces). The results of the tests in the form of a-scan and c-scan images of the longitudinal waves showed that without analysis using advanced data analysis algorithms, the results of the ultrasonic testing, in the form of changes in pulse amplitude or ultrasonic wave propagation time, are not sufficient and give too generalized information about the state of the joint. However, Spytek et al. [34] demonstrated the feasibility of using guided elastic waves generated by a laser beam to evaluate adhesive joints. The proposed test method can obtain clear images of layered joints, which are the basis for determining the quality of the connection. Similar studies using non-contact ultrasonic wave generation in the joint area were performed by Liu et al. [35]. The authors demonstrated the feasibility of using the resonant mode to control metal and adhesive debonding in the tested joint. The results of the ultrasonic guided wave testing of multilayer joints made of metals and composites are also available, proving the usefulness of this method in detecting defects in adhesive joints [36]. Bolstad et al. proposed a system for evaluating the quality of adhesive joints operated under harsh conditions (high temperature and pressure) [37]. Despite the advantages of this system, it is not suitable for verifying adhesive joints made of plywood, adhesive and closed profiles made of steel.

There is a lack of examples of test results available in the literature that use a surface (Rayleigh) wave to assess the condition of an adhesive joint. An analysis of recent articles revealed a knowledge gap in the study of adhesive joints used in bus construction. These are joints where it is not possible to use standard ultrasonic testing that is described in the literature (no longitudinal and transverse wave testing of the joint is possible due to the use of a closed profile and plywood, which does not transmit ultrasonic waves). Therefore, the authors of this article filled this gap by proposing their own procedure for evaluating adhesive joints using surface waves. The main scientific objective of the article was to develop a mathematical model to estimate the width of the adhesive path in a plywood-adhesive-closed-profile joint based on selected parameters of the ultrasonic Rayleigh wave propagating along the closed profile through the adhesive joint. The proposed method allows us to verify the width of the adhesive path, which is important in the case of a lack of access to the joint. The proposed method and the developed model have important practical applications and can be used on the bus production line for the quality control of adhesive joints.

## 2. Research on Adhesive Joints

### 2.1. Research Procedure

**Figure 2:**
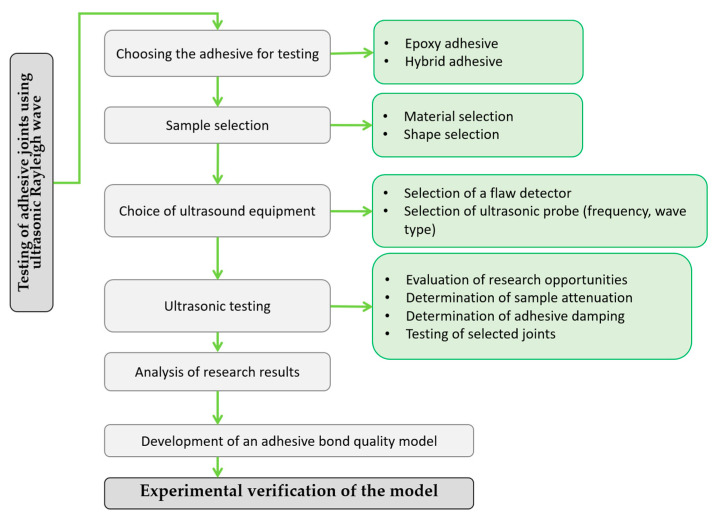
Research procedure used during the experiment.

The experiment consisted of testing the adhesive bond boundary by ultrasonic Rayleigh waves. A digital ultrasonic apparatus, constant coupling between the ultrasonic head and different adhesives were used. All this research work was conducted based on the test procedure shown in Figure 2.

The testing procedure begins with the selection of the adhesive, which should meet all the requirements of bus manufacturers. In this case, two adhesives used at the production stage of these vehicles were selected—a hybrid adhesive and an epoxy adhesive. In the next part of the experiment, samples, using materials used in the construction of buses, were produced. Earlier studies allow us to select an ultrasonic flaw detector and ultrasonic heads with a specific frequency that will allow the wave to propagate around the perimeter of the closed profile. In addition, the frequency of the wave and the energy of the ultrasonic beam must guarantee the passage of the wave through the joint area—the wave must not be attenuated in the joint. In the following section, tests were performed to monitor changes in the attenuation of the ultrasonic wave beam depending on the path width of the applied adhesive. This facilitated the determination of a mathematical equation describing the relationship between the attenuation of the Rayleigh wave and the width of the adhesive path. The width of the adhesive path on a closed profile is very important, as it determines the strength of the joint, especially when it covers large areas of components with a relatively small number of places with applied adhesive.

### 2.2. Materials and Methods

**Figure 3:**
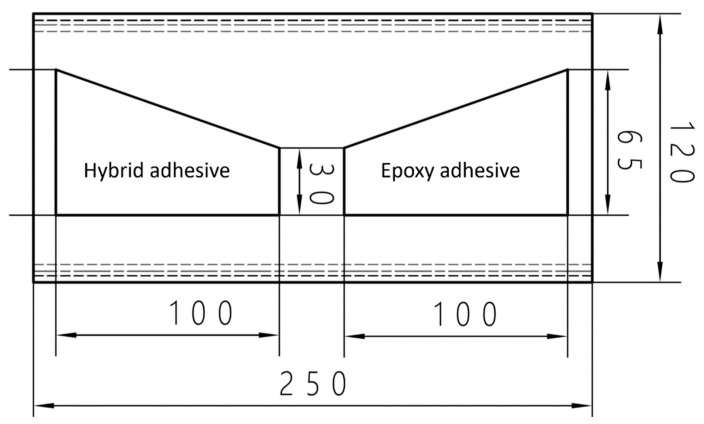
Schematic view of the sample with the main dimensions in mm.

**Figure 4:**
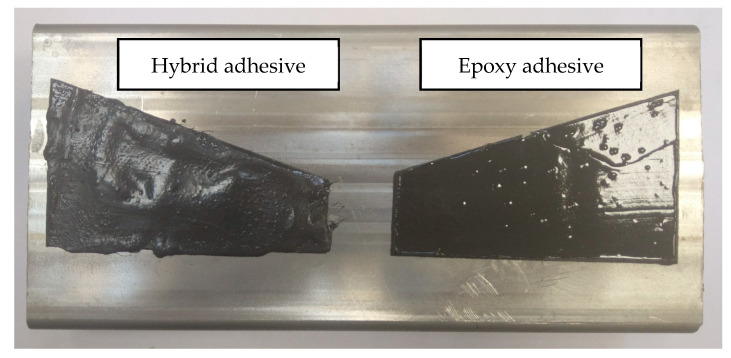
View of the sample with different types of adhesives applied.

Two different types of adhesives were chosen for the study. Both are used in the automotive industry. CX80 Poland hybrid adhesive (CX80, Chotow, Poland) and 3M DP 490 epoxy adhesive (3M company, Maplewood, MN, USA) were selected for the research. The adhesives were applied to steel profiles made of 1.4003 steel. This is a steel with increased corrosion resistance and is used for bus frames. The use of profiles with different cross sections from 40 × 30 × 2 mm to 120 × 40 × 4 mm was considered. It was decided to use the largest profile used for the lower part of the bus frame (120 × 40 × 4 mm) because it is the most difficult to test under manufacturing conditions. The sample was prepared according to the dimensions presented in Figure 3 and is shown in Figure 4.

**Figure 5:**
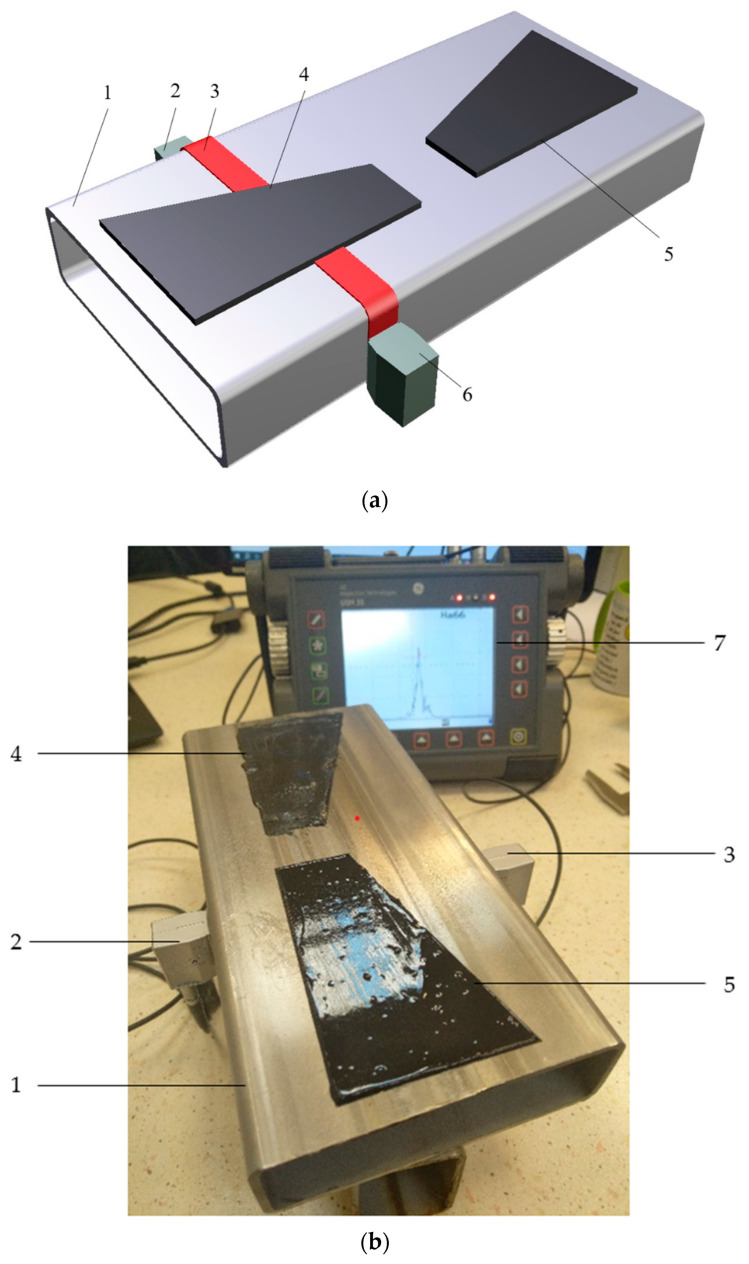
Measurement system: (**a**) scheme: 1—closed profile of 120 × 40 × 4 mm; 2—transmitting head; 3—Rayleigh wave, 4—hybrid adhesive; 5—epoxy adhesive; 6—receiving head; 7—ultrasonic flaw detector, and (**b**) view of the measuring system.

Adhesive joints used in the construction of buses are not possible to test due to the strong damping of the materials used in its construction, namely plywood. The upper part of the joint is plywood, while the lower part has a steel closed profile. Ultrasound will not be transmitted by the air in the closed profile, and the use of an additional coupling medium inside the closed profile is costly and difficult to implement. To evaluate such an adhesive joint, it was decided to use ultrasonic Rayleigh waves. The test setup is shown in Figure 5.

The study used surface wave ultrasonic probes with a frequency of 4 MHz and an 8x9 mm transducer (General Electric, Krautkramer, Boston, MA, USA). It was decided to use such heads because at a frequency of 4 MHz, a relatively low attenuation of the wave and, at the same time, a high resolution of the ultrasonic signal was obtained. Tests were also carried out for probes with a frequency of 10 MHz, but at this frequency the attenuation was so strong that no pulses were obtained on the screen of the ultrasonic flaw detector. The heads were placed opposite each other. A USM35XS GE (General Electric, Krautkramer, Boston, MA, USA) digital ultrasonic flaw detector was used in the research. The measurements of the ultrasonic pulse amplitude were made in different positions of the ultrasonic heads. The tests were carried out with the following ultrasonic flaw detector settings:
• Wave velocity3200 m/s,• Wave amplification70 dB,• P-Delay27.86 µs• Probe center12 mm• Powerlevel

First, the attenuation of a 4 MHz ultrasonic wave in a steel profile (without adhesive) was determined. For this purpose, 30 ultrasonic amplitude height measurements were taken each for different distances between the probes. The measurements were conducted for distances between the centers of the ultrasonic heads of 75, 125 and 175 mm. The determination of the attenuation coefficient in the adhesive material was done based on Equation (1)

(1) $$\alpha =\frac{20}{l}\cdot log\left(\frac{H_{1}}{H_{2}}\right)$$

where H_1_, H_2_ are the percentage height (the amplitude value) of the pulse for two adhesive widths and l is the difference in distance between probes.

The amplitude of the ultrasonic wave measurements were carried out on the prepared sample and the results were observed on the screen of an ultrasonic flaw detector. Measurements were taken for various widths of the adhesive layer (from 35 to 60 mm). In the preliminary tests, the number of repetitions to be performed for one measurement set of the ultrasonic transducer was determined. For this purpose, 50 measurements were carried out for one set of the heads and one width of the adhesive.

The gain of the ultrasonic wave was 70 dB. Measurements were taken continuously, moving both ultrasonic heads along the side surfaces of the steel profile. In preliminary tests, the measurements were repeated ten times.

**Figure 6:**
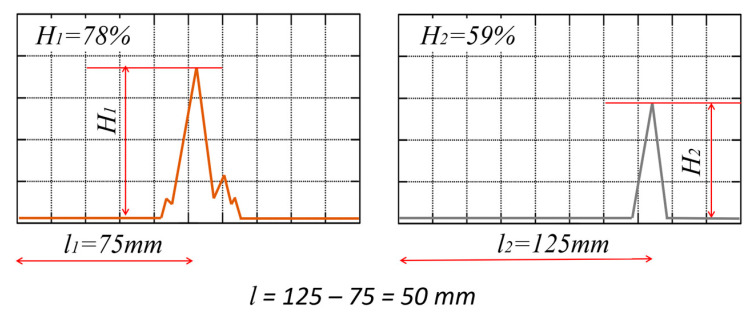
View of the surface wave amplitude measurement.

The scheme of verification and measurement of the ultrasonic surface wave pulse amplitude, propagating in the closed profile along the adhesive-steel connection boundary, is shown in Figure 6.

**Figure 7:**
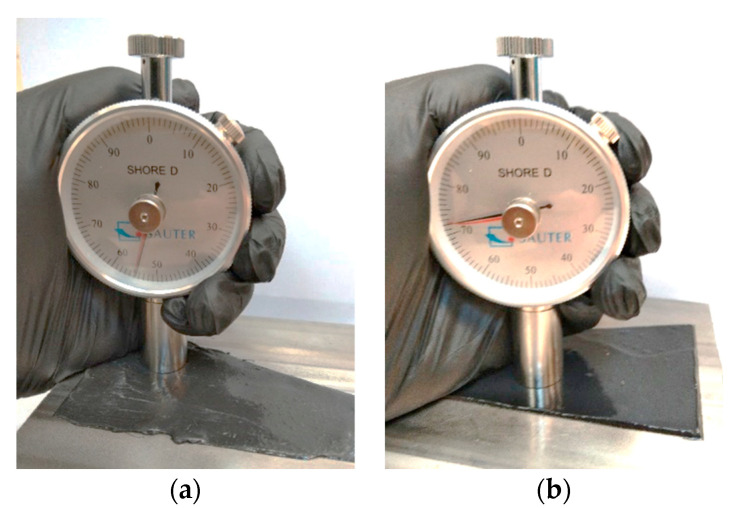
Hardness measurements: (**a**) hybrid adhesive, and (**b**) epoxy adhesive.

Additionally, adhesive hardness measurements were carried out using a Shore hardness tester. Measurements were carried out in various areas of the connections, and repeated ten times. The hardness of the adhesive was studied to see if it would affect the attenuation of the ultrasonic waves. Measuring the hardness of the glue will enable the selection of an ultrasonic model for testing the width of the adhesive path. The hardness test view is shown in Figure 7.

## 3. Results Analysis and Discussion

In the first stage of the research, measurements of the echo amplitude of the ultrasonic surface waves were made for three different distances between the heads, without applying an adhesive. The results of these measurements are summarized in Table 1.

The results summarized in above table clearly show that the greater the distance between the ultrasonic heads, the smaller the amplitude of the ultrasonic wave pulse. This is related to the damping of this wave and the damping coefficient, the value of which was determined in the next step. The average damping coefficient α for only a steel profile (without adhesive) was determined, and was equal to 0.026 dB/mm. In the following part of the research, 50 measurements were performed (results presented in Table 2) and on their basis, the number of measurements for one position of the ultrasonic heads was determined (for one width of the adhesive path applied to the closed profile). The number of measurements was determined based on Equation (2), for a measurement accuracy of 1% of the amplitude height. The determined value was 10 ultrasonic measurements for each selected width of the adhesive path applied to the closed profile.

(2) $$n=\frac{t_{0.05}^{2}\cdot \sigma }{d^{2}}$$

where n is the number of measurements, σ is the standard deviation, and d is the expected accuracy.

The results of the adhesive hardness measurements are presented in Table 3. No significant changes in hardness were found in the area of the tested joints. Nevertheless, the hardness results of the adhesives are important from the point of view of damping the ultrasonic wave propagating through the steel-adhesive connection.

**Figure 8:**
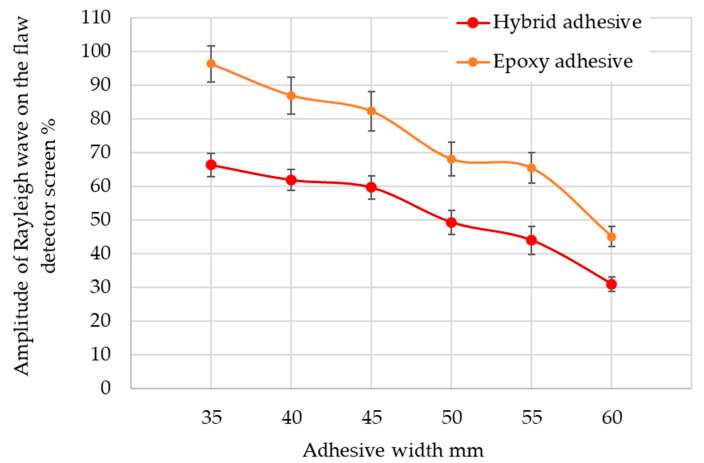
Change of amplitude height depending on the width of the adhesive path.

The results of the pulse amplitude’s average value obtained from the flaw detector screen, taking into account the adhesive path width, are shown in Figure 8. It can be seen that as the width of the adhesive path changes, the pulse height obtained on the flaw detector screen decreases.

The ultrasonic wave damping coefficient at the joint boundary was determined for both types of adhesives. For both adhesives, the value of the ultrasonic wave damping coefficient was similar and was about 0.264 dB/mm, which was much higher than the damping coefficient of the steel profile, where it was equal to 0.026 dB/mm. Taking into account the test results obtained in the figure above, the relationship (3) and (4) was determined. This relationship describes the width of the adhesive path applied to the closed profile (steel sheet) depending on the amplitude (height) of the ultrasonic Rayleigh wave pulse.

W_H_ = −0.013X^2^ + 0.5791X + 54,363 (3)

W_E_ = −0.0336X^2^ + 1.275X + 91,687 (4)

where X is the pulse height on the ultrasonic flaw detector screen, W_H_ is the hybrid adhesive path width and W_E_ is the epoxy adhesive path width.

**Figure 9:**
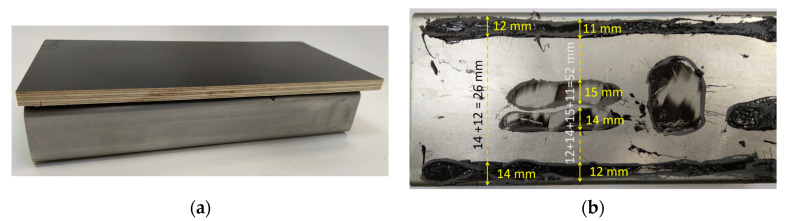
Test sample: (**a**) after bonding during the test, and (**b**) after tearing to verify the results.

**Figure 10:**
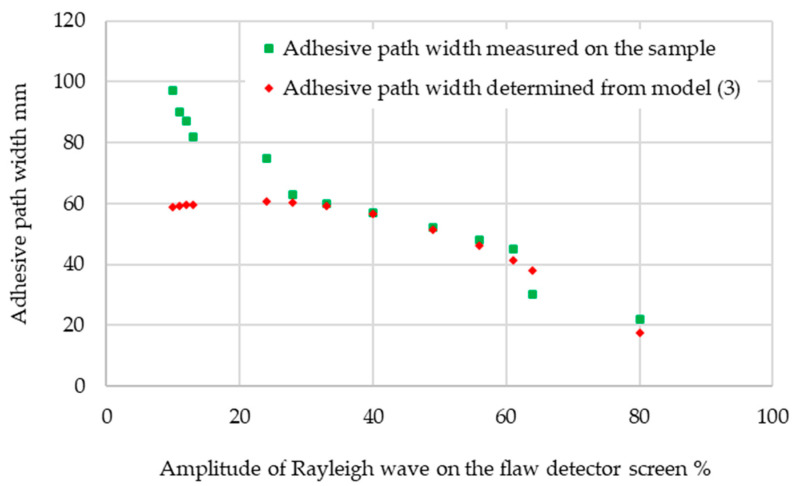
Verification of the developed model in Equation (3).

In the next step of the experiment, the determined relationship describing the dependence of the amplitude of the ultrasonic surface wave pulse on the width of the adhesive path was verified. For this purpose, a sample with different adhesive path widths was prepared, and is shown in Figure 9. The sample was made from a 120 × 30 × 4 mm steel profile with a length of 250 mm. This is the kind of profile that is used in the production of city buses. Hybrid glue was applied in such a way that there was a different width of the glue path. Then, such plywood as is used for the floor was applied. After the joint was constituted, measurements were taken using surface wave heads. After the measurements were completed, the plywood was separated from the glue and the width of the glue path was measured, which allowed verification of the model. Then, the ultrasonic Rayleigh wave pulse amplitudes were determined in different places of the sample and the width of the adhesive path was calculated using Equation (3). The values determined in this way were compared with the situation presented in Figure 10.

The average difference between the measurements for the hybrid adhesive and the values calculated based on the model is 12%. The proposed model is characterized by high accuracy in the verification of the width of the adhesive path from about 20 mm to 65 mm, which corresponds to the amplitude of the pulses on the flaw detector screen, from about 25 to 80 percent of the screen height. To effectively test the adhesive joints of smaller widths, it is necessary to use ultrasonic heads at a higher frequency, e.g., 8 MHz. To test joints with an adhesive path width exceeding 65 mm, it is better to use heads with a lower frequency, e.g., 1 or 2 MHz. The issue of adhesive damping is an important issue, considered from various aspects. In [38], the damping of adhesives used to protect elements undergoing machining against vibrations was investigated. These works were carried out for relatively low vibration frequencies (up to 100 kHz) and it was possible to reduce the unfavorable vibrations by 58% using polyurethane adhesive. Research was carried out on the influence of adhesives on the damping of lap-bonded samples [39]. It was found that the attenuation due to adhesives in the vibrating structures is small and much less than in the case of adhesive alone. It should not be expected that bonding can be used to increase the damping of the structure. It should be emphasized that the research included different samples than in this study. Work in the area of ultrasonic assessment of adhesive joints was also carried out using ultrasonic guided waves [40]. A relatively low frequency of 500 kHz was used, and the results of simulation tests were verified with laboratory measurements. The authors stated that the simulations are consistent with the measurement results. The methods used by researchers do not enable the testing of connections such as those made on a closed profile. The test object presented in this article is an object with low susceptibility for ultrasonic testing. It is difficult, or even impossible, to implement ultrasonic longitudinal waves. The results of the research confirm that the proposed method will make it possible to inspect adhesive joints where it has not been possible so far. These are mainly such objects as the bodies of passenger-carrying vehicles (buses, streetcars, railroad cars), that is, such objects where the plating is adhered to steel frames. Since the plating and floors are made from different materials (skeleton, plastic, sheet metal), the proposed inspection method is particularly relevant. Research on adhesive joints using various ultrasonic waves is a promising field in materials and industrial engineering [41,42]. However, the use of the ultrasonic method of transmitting an ultrasonic wave beam was not used, and the proposed surface wave testing technique facilitates the assessment of the width of the adhesive path under conditions of limited access to the joint. Research available in the literature focuses on understanding the impact of various ultrasonic parameters, such as the frequency and amplitude of the ultrasonic waves, on the assessment of the quality and strength of an adhesive joint [43]. As a result, the approach used by the authors not only expands the knowledge about the use of ultrasonic methods in the process of assessing the quality of adhesive joints, but above all allows for the verification of these joints in vehicle production conditions. This is an undoubted advantage of the proposed approach to controlling adhesive connections currently used in manufactured buses.

## 4. Conclusions

The research results confirmed that ultrasonic Rayleigh waves allow us to determine the adhesive path regardless of the type of adhesive. A section of a bus floor was tested. The tests used two different adhesives—a hybrid adhesive and an epoxy adhesive. The tests were conducted on a 120 × 40 × 4 mm steel profile using ultrasonic surface wave heads. The study also examined the hardness of the two adhesives, which differed significantly. It was found that the hardness of the hybrid adhesive was 55 Shore on the D scale, while that of the two-component epoxy adhesive was 73 Shore on the D scale. It was also verified that it was possible to detect areas with varying adhesive path widths. The proposed method can find application where adhesive joints are used to join closed sections.

Directions for further research should include the use of amplitude–frequency analysis in assessing the width of the adhesive path. Moreover, the authors plan to perform similar tests not only for different widths but also for different thicknesses of adhesives applied to the closed profile. As a result, a database of mathematical models will be created that will allow us to estimate the surface on which the adhesive was applied in relation to the entire covering of a different bus body element.

## Figures and Tables

**Table 1:** Compilation of the measurement results to calculate the damping coefficient α.

No	Distance mm	Average Pulse Height%	Standard Deviation	Half Confidence Interval
1	75	75.567	1.116	1.896
2	125	65.233	1.542	2.620
3	175	56.667	1.619	2.751

**Table 2:** Results of the preliminary measurements of the ultrasonic Rayleigh wave parameters.

No	H [%]	No	H [%]	No	H [%]	No	H [%]	No	H [%]
1	51	11	51	21	54	31	56	41	52
2	51	12	50	22	53	32	55	42	51
3	49	13	52	23	55	33	53	43	53
4	52	14	51	24	56	34	53	44	53
5	52	15	52	25	52	35	53	45	51
6	50	16	54	26	53	36	55	46	53
7	51	17	52	27	53	37	51	47	55
8	51	18	54	28	53	38	55	48	51
9	53	19	51	29	53	39	54	49	55
10	51	20	53	30	54	40	52	50	50

**Table 3:** Hardness measurement results.

	Medium Shore Hardness on the D Scale	Standard Deviation	Half Confidence Interval
Hybrid adhesive	55.4	1.066	2.411
Epoxy adhesive	72.8	0.916	2.073

## Data Availability

The data presented in this study are available on request from the corresponding author.
